# Long-term follow-up of tislelizumab combined with anlotinib in a single patient with metastatic type B3 thymoma: a case report

**DOI:** 10.3389/fonc.2026.1856689

**Published:** 2026-08-19

**Authors:** Ting Jiang, Chen Gao, Fangmi Ding, Shuyi Chen, Qunwei Chen

**Affiliations:** 1 Department of Oncology, The First Affiliated Hospital of Zhejiang Chinese Medical University (Zhejiang Provincial Hospital of Chinese Medicine), Hangzhou, China; 2 Department of Radiology, The First Affiliated Hospital of Zhejiang Chinese Medical University (Zhejiang Provincial Hospital of Chinese Medicine), Hangzhou, China

**Keywords:** anlotinib, case report, follow-up, thymoma, tislelizumab

## Abstract

**Background:**

Thymoma is a rare epithelial malignancy. Type B3 thymoma confers poor prognosis largely owing to limited effective systemic therapies. Although immune checkpoint inhibitors and multi-target tyrosine kinase inhibitors have shown promising therapeutic efficacy in multiple solid tumors, yet their application in thymoma remains insufficiently investigated.

**Case presentation:**

A middle-aged woman with chest tightness and dyspnea was diagnosed with metastatic type B3 thymoma involving multiple mediastinal, hilar, and pulmonary lesions. After progression on first-line platinum-based chemotherapy, she was treated with tislelizumab and anlotinib. The combination therapy achieved a marked objective response, resulting in tumor regression, with over 30 months of progression-free survival and a maintained good quality of life.

**Conclusion:**

This case suggests that the combination of tislelizumab and anlotinib may exhibit promising antitumor activity in patients with metastatic type B3 thymoma. However, the therapeutic efficacy of this regimen cannot be established based on a single case. Therefore, further prospective studies with larger, well-controlled clinical cohorts are required to validate its safety and efficacy.

## Introduction

Thymoma is a rare mediastinal neoplasm originating from the epithelial cells of the thymus. Histopathologically, it is classified into five distinct subtypes, among which type B3 thymoma exhibits the most aggressive biological behavior and is associated with an unfavorable prognosis and reduced survival outcomes (1, 2). According to the National Comprehensive Cancer Network (NCCN) guidelines, complete surgical resection remains the preferred treatment whenever feasible. For patients with unresectable disease, platinum-based chemotherapy is recommended as the standard first-line systemic treatment. However, therapeutic options following disease progression on platinum-containing regimens remain extremely limited. Current evidence supporting second-line therapies is derived primarily from small-scale and phase II clinical trials, providing insufficient high-quality data to guide clinical decision-making (3–5). The lack of large, randomized phase III studies largely reflects the rarity of thymoma and the inherent difficulties in recruiting adequate patient populations. In recent years, programmed cell death protein-1 (PD-1) immune checkpoint inhibitors (ICIs) and multi-target tyrosine kinase inhibitors (mTKIs) have demonstrated remarkable therapeutic efficacy and have been approved for the treatment of a variety of solid malignancies. Nevertheless, their clinical utility in thymoma, particularly in patients with advanced type B3 disease, remains largely undefined. Here we present the case of a patient with metastatic type B3 thymoma who achieved prolonged survival and manageable treatment-related toxicity with a combination of tislelizumab, an anti-PD-1 monoclonal antibody, and anlotinib, an mTKI with anti-angiogenic activity, after experiencing disease progression following platinum-based chemotherapy.

## Case description

**Figure 1:**
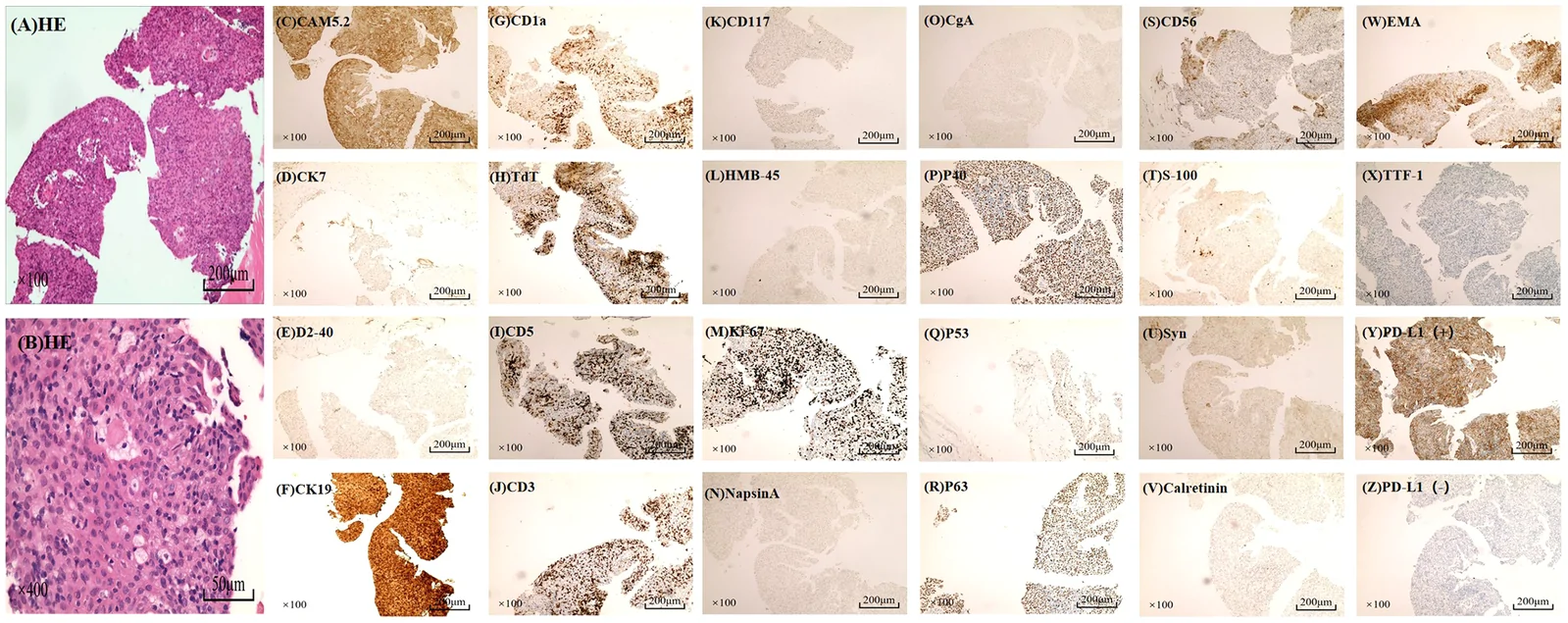
HE and IHC staining of thymoma tissues with magnification scale bar and magnification label. **(A)** HE staining (×100). **(B)** HE staining (×400). The IHC staining results were validated using internal controls and are shown as follows: **(C)** CAM5.2 (+), **(D)** CK7 (-), **(E)** D2-40 (positive in lymphatic vessels), **(F)** CK19 (+), **(G–J)** CD1a, TdT, CD5, and CD3 (positive in background cells), **(K)** CD117 (-), **(L)** HMB-45 (-), **(M)** Ki-67 (50%+), **(N)** NapsinA (-), **(O)** CgA (-), **(P)** P40 (+), **(Q)** P53 (10%+), **(R)** P63 (+), **(S)** CD56 (focally positive), **(T)** S-100 (-), **(U)** Syn (-), **(V)** calretinin (-), **(W)** EMA (+), and **(X)** TTF-1 (-), all at ×100 magnification. **(Y)** PD-L1 (TPS ≥50%) and **(Z)** the corresponding valid negative control (×100). HE, hematoxylin and eosin; IHC, immunohistochemistry; PD-L1, programmed cell death-ligand 1; TPS, tumor proportion score.

A 52-year-old woman presented to our institution on December 28, 2022 with complaints of chest tightness and shortness of breath. Chest computed tomography (CT) revealed multiple masses located in the para-aortic region of the upper mediastinum, the left pre-cardiac antero-middle mediastinum, the left hilar region, and the left lung parenchyma (**Supplementary Figures 1A–D**). Whole-body positron emission tomography/CT subsequently confirmed these findings. Physical examination demonstrated decreased breath sounds over the left lung. The patient exhibited no clinical manifestations suggestive of myasthenia gravis, including ptosis, diplopia, limb weakness, dysphagia, exertional dyspnea, or fatigability. She also had no history of autoimmune diseases, such as rheumatoid arthritis or thyroiditis. The patient was a homemaker who routinely performed household activities. She had never smoked and reported no occupational exposure to dust, chemical toxins, ionizing radiation, or other known carcinogens. Mass puncture biopsy was performed. Histopathological examination established the diagnosis of stage IVA type B3 thymoma according to the Masaoka–Koga staging system and the World Health Organization histological classification. Hematoxylin and eosin staining demonstrated that nests of atypical cells were present within hyperplastic fibrous tissues, with stromal lymphocytes visible (**Figures 1A, B**). Immunohistochemical analysis with internal controls revealed the following profile: CAM5.2 (+), CK7 (-), D2-40 (positive in lymphatic vessels), CK19 (+), CD1a, TdT, CD5, and CD3 (positive in background cells), CD117 (-), HMB-45 (-), Ki-67 (50%+), NapsinA (-), CgA (-), P40 (+), P53 (10%+), P63 (+), CD56 (focally positive), S-100 (-), Syn (-), calretinin (-), EMA (+), and TTF-1 (-) (**Figures 1C–X**). Programmed death-ligand 1 (PD-L1) expression was detected via the VENTANA SP263 assay on the BenchMark ULTRA platform. Tumor proportion score (TPS) refers to the proportion of tumor cells with membranous PD-L1 staining. This tumor showed high PD-L1 expression (TPS≥50%), and positive/negative controls confirmed acceptable staining quality (**Figures 1Y, Z**). All histopathological results underwent standardized, dual, independent, blinded review: initial assessment by an attending pathologist, with final confirmation by a chief pathologist.

The patient initially refused antitumor therapy because of her fear of chemotherapy. However, a chest CT scan performed on January 30, 2023 demonstrated an increase in the size of all tumor lesions (**Supplementary Figures 1E–H**), after which she agreed to undergo chemotherapy. She subsequently received six cycles of chemotherapy between January 31 and June 1, 2023, consisting of cyclophosphamide (700 mg, day 1), epirubicin (120 mg, day 1), and cisplatin (35 mg, days 1 and 2), administered every 3 weeks. Serial imaging evaluations performed on March 15, May 2, and June 26, 2023 demonstrated mild regression of the target lesions. According to the Response Evaluation Criteria in Solid Tumors (RECIST) version 1.1, the therapeutic response at each assessment was classified as stable disease (SD) compared with the pre-treatment scan dated on January 30, 2023 (**Supplementary Figures 1I–T**).

**Figure 2:**
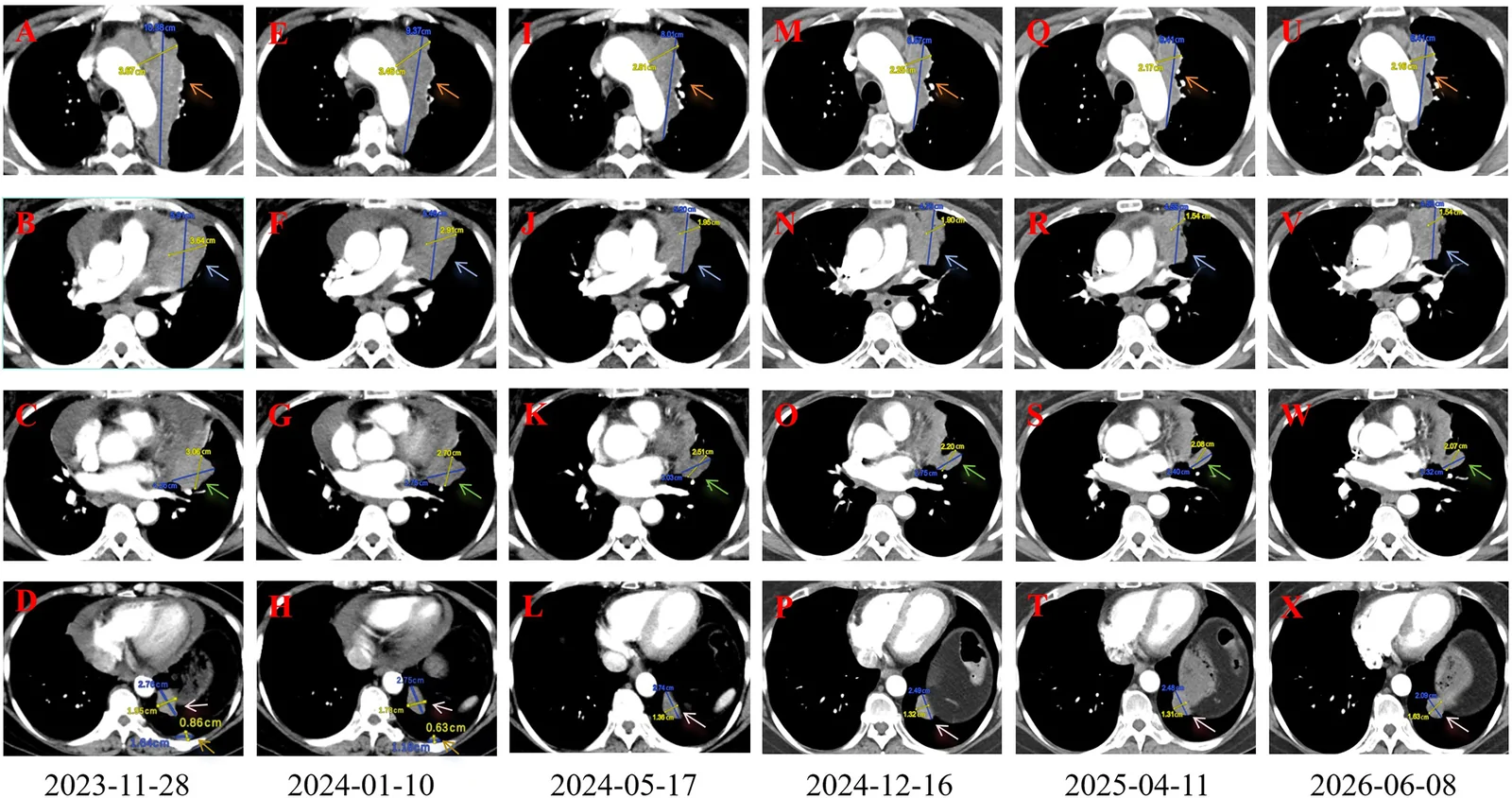
Dynamic images of thymoma lesions during tislelizumab plus anlotinib therapy. **(A–D)** All lesions involving the para-aortic region of the upper mediastinum, left pre-cardiac antero-middle mediastinum, left hilum region, and left intrapulmonary parenchyma enlarged and a new left pleural lesion emerged, resulting in an evaluation of progressive disease. **(E–H)** After two treatment cycles, all tumor lesions showed mild shrinkage and were evaluated as stable disease. **(I–L)** After an additional six treatment cycles, the left pleural lesion completely resolved while the remaining lesions showed slight shrinkage, resulting in an overall evaluation of stable disease. **(M–P)** After an additional 10 treatment cycles, all lesions showed further shrinkage, with the overall response remaining a partial response. **(Q–T)** All lesions shrank further after another five cycles, remaining a partial response. **(U–X)** After an additional 19 treatment cycles, the left intrapulmonary lesion showed slight enlargement, whereas the overall disease status remained a partial response. 
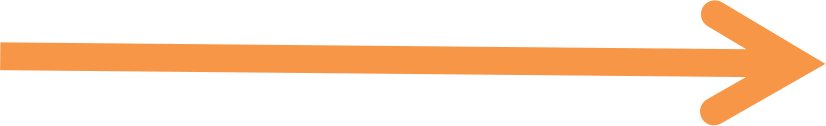
 The lesion in the para-aortic region of the upper mediastinum; 
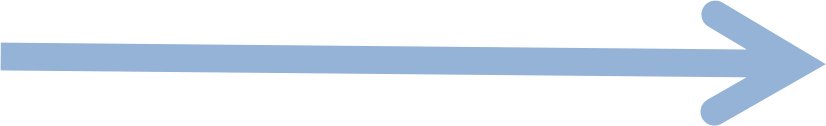
 The lesion in the left pre-cardiac antero-middle mediastinum region; 
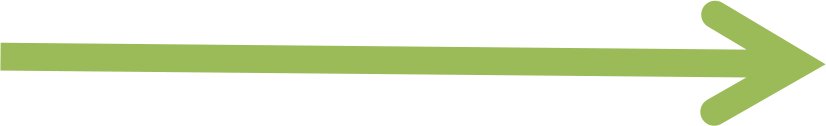
 The lesion in the left hilum region; 
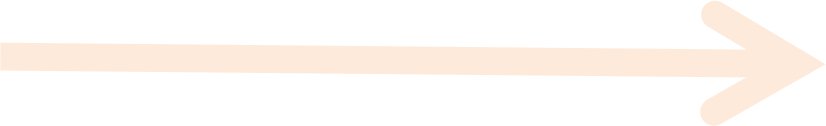
 Intrapulmonary lesion within left lung; 
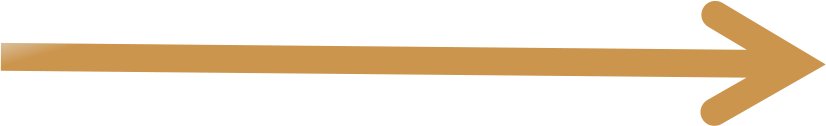
 The lesion on left pleura.

On November 28, 2023, a follow-up chest CT scan demonstrated enlargement of all previously identified lesions located in the para-aortic region of the upper mediastinum, the left pre-cardiac antero-middle mediastinum, the left hilum region, and the left intrapulmonary parenchyma, with the appearance of a new pleural lesion on the left side, indicating progressive disease (PD) according to the RECIST version 1.1 (**Figures 2A–D**). In view of the tumor’s high PD-L1 expression and the absence of other effective second-line treatment options, combination therapy was initiated on November 29, 2023. The treatment regimen consisted of tislelizumab (200 mg, administered intravenously on day 1) in combination with anlotinib (10 mg, administered orally once daily on days 1–14), with each treatment cycle repeated every 21 days.

After two cycles of treatment, a follow-up chest CT performed on January 10, 2024 demonstrated mild shrinkage of all tumor lesions, corresponding to SD according to RECIST version 1.1 (**Figures 2E–H**). The same combination regimen was subsequently continued for an additional six cycles. Chest CT imaging obtained on May 17, 2024 showed complete resolution of the left pleural mass and a further reduction in the size of the remaining lesions. Nevertheless, the overall treatment response at this time point was assessed as SD according to RECIST version 1.1 (**Figures 2I–L**). The patient then received 10 additional cycles of the same regimen, and a chest CT performed on December 16, 2024 revealed marked regression of all tumor lesions, consistent with a partial response (PR) based on RECIST version 1.1 (**Figures 2M–P**). Treatment with tislelizumab combined with anlotinib was subsequently continued for another five cycles. Follow-up imaging on April 11, 2025 demonstrated further shrinkage of all lesions, with the overall response remaining PR (**Figures 2Q–T**). Accordingly, the same treatment regimen was maintained for an additional 19 cycles. The most recent chest CT, performed on June 8, 2026, revealed a slight enlargement of the left intrapulmonary metastatic lesion; however, the overall therapeutic response continued to meet the criteria for PR (**Figures 2U–X**).

At the time of this case report, the patient had successfully completed a total of 44 cycles of treatment with tislelizumab plus anlotinib, with the most recent cycle administered on July 6, 2026. No evidence of new metastatic lesions was detected. Progression-free survival (PFS) was calculated from the initiation of tislelizumab plus anlotinib on November 29, 2023. As of July 2026, the patient had achieved a PFS of more than 30 months.

**Figure 3:**
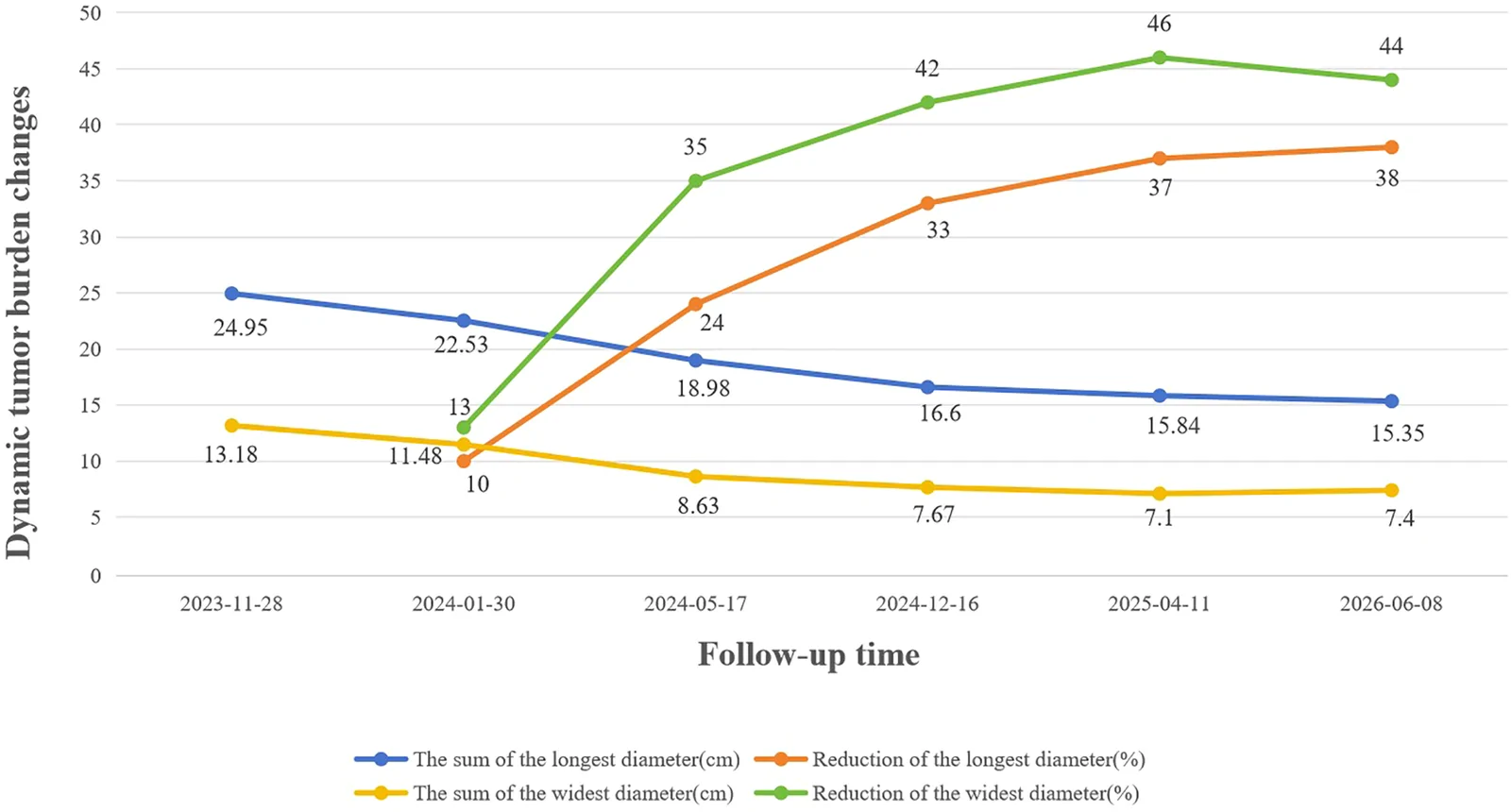
The line graph of tumor burden changes following Tislelizumab combined with Anlotinib.

**Figure 4:**
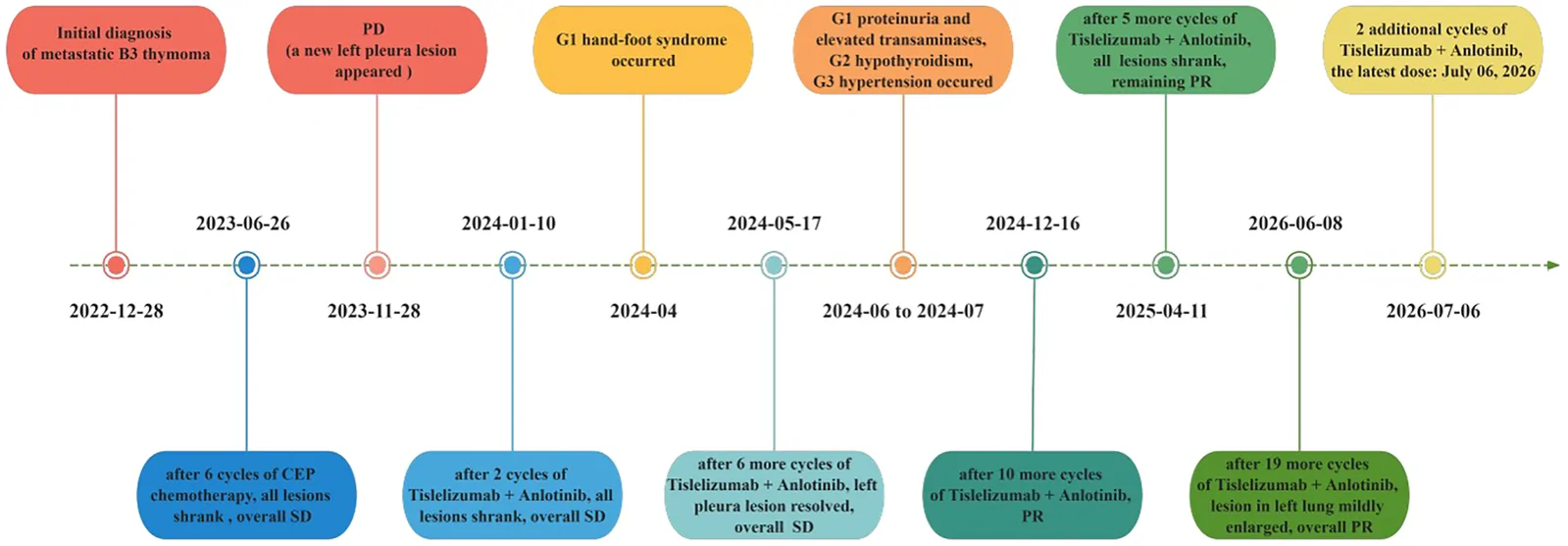
Flowchart of the patient’s entire treatment course. CEP, cyclophosphamide + epirubicin + cisplatin; SD, stable disease; PD, progressive disease; PR, partial response.

Although RECIST version 1.1 evaluates target lesions based on their longest diameter, a concomitant reduction in the widest diameter of the lesions was also observed in this case. For greater illustrative clarity, both the longest and widest diameters of representative lesions are annotated in **Figure 2**; **Supplementary Figure 1**; **Supplementary Tables 1**, **2** also summarized the serial changes in the longest and widest diameters of each lesion during chemotherapy and subsequent tislelizumab plus anlotinib combination therapy, along with full records of efficacy assessment. Serial magnified CT panels of lesions during combination therapy, annotated with lesion diameters and directional arrows as shown in **Figure 2**, are available in the **Supplementary Data**. **Figure 3** presents a line graph depicting the dynamic changes in lesion diameters and the corresponding percentage reduction throughout the combination therapy. **Figure 4** illustrates the patient’s treatment timeline from the initial diagnosis of thymoma. Overall, following combination therapy with tislelizumab and anlotinib, the left pleural lesion completely resolved, whereas all lesions achieved a best overall response of PR and have remained in PR to date.

According to the Common Terminology Criteria for Adverse Events version 6.0, the patient experienced grade 1 hand–foot syndrome and grade 1 elevations in liver transaminases (alanine aminotransferase, 104 U/L; aspartate aminotransferase, 63 U/L) during treatment with tislelizumab plus anlotinib. These adverse events resolved following appropriate management. Grade 2 hypothyroidism was observed, which was successfully managed with daily oral levothyroxine. In addition, at cycle 12 of the combination therapy, the patient developed grade 3 hypertension with concomitant grade 1 proteinuria. With daily oral amlodipine and adherence to a low-sodium, high-quality, low-protein diet, she achieved well-controlled blood pressure and persistent grade 1 proteinuria. The serum albumin and creatinine levels were normal, without peripheral edema. No other treatment-related adverse events were observed. Neither temporary interruption, dose reduction, nor permanent discontinuation of tislelizumab or anlotinib was required. Detailed information on all treatment-related adverse events is summarized in **Supplementary Table 3**. The patient was highly satisfied with the treatment and has maintained a good quality of life.

## Discussion

This case report describes a single patient with metastatic type B3 thymoma who achieved long-term survival benefit following treatment with tislelizumab in combination with anlotinib after failure of cisplatin-based chemotherapy. Nevertheless, as this is a single-case observation, it can only generate preliminary clinical hypotheses and cannot establish the efficacy of tislelizumab plus anlotinib for metastatic type B3 thymoma. Large-scale prospective controlled studies are required to validate these preliminary findings.

According to the NCCN guidelines for thymomas and thymic carcinomas, therapeutic options in the second-line setting remain limited and include everolimus, gemcitabine, capecitabine, and pemetrexed (3–5). The available evidence supporting these agents is derived primarily from phase II clinical trials, reflecting their limited clinical efficacy. Conducting phase III randomized trials in thymoma is particularly challenging because of the rarity of the disease. In recent years, increasing attention has been directed toward immunotherapy as a potential treatment strategy for advanced thymoma. In a phase I clinical trial evaluating avelumab, four of seven patients with thymoma achieved an objective response, corresponding to an objective response rate (ORR) of 57% (6). Subsequently, a single-arm phase II study of pembrolizumab reported an ORR of 28.6% and a median PFS (mPFS) of only 6.1 months among seven patients with thymoma (7). These findings highlight the limited efficacy of currently available therapies and underscore the urgent need for more effective treatment strategies for advanced thymoma.

In recent years, combination therapy consisting of ICIs and mTKIs has emerged as a promising treatment strategy, demonstrating synergistic antitumor activity across a variety of solid tumors. A single-arm phase II trial evaluated tislelizumab combined with anlotinib as first-line therapy for patients with advanced pulmonary sarcomatoid carcinoma and reported an ORR of 55.17% and a disease control rate (DCR) of 96.55%. The median overall survival (mOS) and mPFS were 14.37 and 9.4 months, respectively. Furthermore, the combination demonstrated a favorable safety profile, with most treatment-related adverse events being manageable (8). Similarly, a phase III randomized controlled trial in patients with advanced renal cell carcinoma demonstrated that toripalimab plus axitinib was superior to sunitinib, achieving a significantly longer mPFS (18.0 vs. 9.8 months), while the mOS had not yet been reached compared with 26.8 months in the sunitinib group. The combination regimen also produced a markedly higher ORR (56.7% vs. 30.8%) (9). In addition, the CARES-310 trial demonstrated that patients with advanced hepatocellular carcinoma treated with camrelizumab plus rivoceranib achieved significantly prolonged survival and a higher ORR than those receiving sorafenib (10). Despite these encouraging findings in other malignancies, the application of immunotherapy combined with mTKIs in thymic tumors remains at an exploratory stage. Two single-arm phase II clinical trials have investigated the efficacy and safety of combining ICIs with antiangiogenic agents in patients with platinum-refractory advanced type B3 thymoma or thymic carcinoma. In the CAVEATT trial, avelumab (anti-PD-L1) plus axitinib was administered to previously treated patients, including those with prior exposure to antiangiogenic therapy. The study reported an ORR of 34%, a DCR of 91%, an mPFS of 7.5 months, and an mOS of 26.6 months. Among the five patients with pure or mixed type B3 thymoma, two achieved PR, while the remaining three maintained SD. Most treatment-related adverse events were mild (11). The PECATI trial, led by Jordi Remon, likewise evaluated pembrolizumab (anti-PD-1) in combination with lenvatinib in patients who had previously received chemotherapy but had not been treated with antiangiogenic agents. The study reported an ORR of 23.3%, a DCR of 93%, an mPFS of 14.9 months, and an unreached mOS at the time of data cutoff. Most treatment-related toxicities were mild to moderate in severity (12). In contrast to these prospective studies with systematically collected efficacy data, our report describes a single patient with metastatic type B3 thymoma exhibiting high PD-L1 expression who experienced disease progression following platinum-based chemotherapy. Because of the high level of PD-L1 expression and the potential synergistic effect of combining targeted therapy with immunotherapy, the patient was subsequently treated with tislelizumab (anti-PD-1) plus anlotinib without prior exposure to either immunotherapy or antiangiogenic therapy. The patient achieved a PR, with PFS exceeding 30 months to date. During treatment, the patient experienced manageable grade 3 adverse events as well as several mild grades 1 to 2 toxicities.

Tislelizumab is a PD-1 inhibitor, with high affinity for PD-1. As a humanized immunoglobulin G4 monoclonal antibody, it attenuates binding to Fc gamma receptors on macrophages, thereby enhancing its antitumor activity (13). Accumulating evidence has demonstrated its favorable therapeutic activity and well-established safety profile across a variety of malignancies (14–16). Clinical studies have also confirmed that anlotinib exhibited significant antitumor activity in a broad spectrum of solid tumors, including lung cancer and hepatocellular carcinoma (17, 18). Its antitumor effects are primarily mediated through the inhibition of vascular endothelial growth factor receptor 2, platelet-derived growth factor receptor-β, and fibroblast growth factor receptor 1, thereby suppressing tumor angiogenesis (19). Preclinical studies have demonstrated that vascular normalization facilitates immune cell infiltration into the tumor microenvironment, alleviates tumor hypoxia, and promotes favorable immunomodulatory remodeling of the tumor microenvironment (20). In addition, thymomas are characterized by high PD-L1 expression, which has been associated with an improved response to immune checkpoint inhibitor therapy (21). Collectively, these biological mechanisms may provide a rationale for the use of combined targeted therapy and immunotherapy in the present patient.

For this patient, who has received combined immunotherapy and targeted therapy for more than 2 years, the rationale for prolonged treatment is summarized as follows. To date, no consensus or absolute guideline recommends limiting combined immunotherapy and targeted therapy to a fixed duration of 2 years. In the KEYNOTE-010 trial, 32.4% (23/71) of patients experienced disease relapse after discontinuing immunotherapy following 2 years of treatment (22). Another clinical study demonstrated that patients who continued treatment beyond 2 years achieved significantly longer mPFS than those who discontinued therapy at the 2-year mark, whereas the discontinuation group exhibited a higher risk of disease progression (23). Similarly, the CAVEATT trial investigating avelumab plus axitinib in patients with metastatic thymic malignancies permitted treatment to continue until disease progression or the occurrence of unacceptable toxicity (11). A real-world retrospective study of patients with advanced renal cell carcinoma further supported the long-term efficacy and acceptable safety of continuing first-line immunotherapy combined with targeted therapy after 2 years of sustained disease control. In that study, 85.7% of patients achieved a PR and 14.3% achieved a complete response (CR), with a 3-year PFS rate of 71.4% and a 5-year OS rate of 58.3%. Prolonged treatment also resulted in deeper tumor responses, with one patient converting from SD to PR and two patients converting from PR to CR. Furthermore, patients who achieved their maximum tumor regression after 24 months experienced superior PFS and OS compared with those whose greatest tumor shrinkage occurred within the first 2 years of therapy (24), suggesting that continued treatment may provide additional clinical benefit. Moreover, several clinical trials involving other malignancies have likewise demonstrated that extending combined immunotherapy and targeted therapy beyond two years is associated with sustained efficacy and a manageable safety profile (8, 18, 25, 26). This patient failed to achieve CR after 2 years of treatment, with persistent residual lesions on imaging showing a shrinking trend. Early treatment discontinuation raises risks of disease progression and severe complications. Moreover, the regimen was well tolerated without intolerable adverse events. Thus, we continued the combination therapy.

Despite the promising therapeutic potential of immunotherapy for thymoma, clinicians must carefully balance its clinical benefits against the risk of immune-related adverse events (irAEs), given the close association between thymoma and autoimmune disorders. Before initiating immune-based treatment, appropriate patient selection is essential, with thorough screening to exclude potential risk factors, particularly pre-existing autoimmune diseases. A large-scale study involving 36,387 patients identified several risk factors for severe irAEs, including age ≥65 years, male sex, early onset of the first immune-related adverse event, and a greater number of adverse events during treatment (27). In addition, it has been reported that elevated C-reactive protein levels, an increased neutrophil-to-lymphocyte ratio, treatment with PD-1 inhibitors, high PD-L1 expression, smoking status, and a favorable therapeutic response are associated with an increased risk of developing irAEs (28). To further reduce the incidence of irAEs and optimize the safety of immunotherapy, additional predictive biomarkers are needed to identify patients who are most likely to benefit from treatment while minimizing the risk of severe toxicity.

Nevertheless, this case report has several limitations. First of all, favorable outcomes in a single case cannot represent the overall efficacy of the combination regimen of tislelizumab and anlotinib. Larger prospective trials are needed to validate its efficacy and safety in wider patient cohorts. In addition, comprehensive genomic testing was not performed, and dynamic changes in the quantitative distribution and functional phenotypes of immune cell subpopulations in peripheral blood or tumor tissue could not be evaluated because the patient declined these investigations owing to their high cost. Consequently, the patient’s genomic landscape and tumor immune microenvironment remain uncharacterized, making it impossible to determine whether the observed therapeutic response was associated with specific molecular or immunological features.

## Conclusion

In conclusion, we report a patient with metastatic B3 thymoma who achieved long-term survival following a combination therapy of tislelizumab and anlotinib after progression on platinum-based chemotherapy. This case only provides preliminary observational signal, and further prospective studies and larger clinical trials are warranted to validate its efficacy and safety.

## Data Availability

The original contributions presented in the study are included in the article/**Supplementary Material**. Further inquiries can be directed to the corresponding author.
