# Supplementary material for: Long-term follow-up of tislelizumab combined with anlotinib in a single patient with metastatic type B3 thymoma: a case report
Source: Front Oncol. 2026 Aug 19;16:1856689. doi: 10.3389/fonc.2026.1856689 (PMC13533702; doi:10.3389/fonc.2026.1856689)
Supplement: Supplementary file 1 [file SupplementaryFile2.docx]

Supplementary material captions

1. Supplementary Data

Serial magnified CT images of thymoma lesions during combination therapy of Tislelizumab and Anlotinib

# 2. **Supplementary Figure and Tables**

## 2.1 Supplementary Figure

**Supplementary Figure 1.**

Dynamic changes in thymoma lesions during chemotherapy with Cyclophosphamide, Epirubicin, and Cisplatin.

(A-D) Baseline lesions were located in para-aortic region of the upper mediastinum, the left pre-cardiac antero-middle mediastinum, left hilum region and the left intrapulmonary region.

(E-H) All lesions enlarged after 1 month.

(I-L) After 2 cycles of chemotherapy, all lesions shrank and were evaluated as stable disease.

(M-P) After an additional 2 cycles of chemotherapy, all lesions showed further shrinkage and were again evaluated as stable disease.

(Q-T) All lesions showed mild shrinkage after 2 more cycles, evaluated as stable disease.

## 2.2 Supplementary Tables

**Supplementary Table 1.**

Response assessment during chemotherapy (Cyclophosphamide+Epirubicin+Cisplatin)

**Supplementary Table 2.**

Response assessment during Tislelizumab combined with Anlotinib treatment

**Supplementary Table 3.**

Adverse events during treatment with Tislelizumab combined with Anlotinib
